# Supplementary material for: Learning with sparse reward in a gap junction network inspired by the insect mushroom body
Source: PLoS Comput Biol. 2024 May 23;20(5):e1012086. doi: 10.1371/journal.pcbi.1012086 (PMC11152299; doi:10.1371/journal.pcbi.1012086)
Supplement: S1 Fig — (A) trained with ‘AM+/OCT’, tested with ‘AON’, (B) trained with ‘AM/OCT+’, tested with ‘AON’, (C) trained with ‘AM+/OCT’, tested with ‘AOF’, (D) trained with ‘AM/OCT+’, tested with ‘AOF’. Left column: the final synaptic strengths. Right column: the change of synaptic strengths. Each line is one connection between two states, colour coded as in the legend. The red vertical lines marks the change of Petri dish. (PDF) [file pcbi.1012086.s001.pdf]

S1 Fig for "Learning with sparse reward in a gap junction network inspired by the insect mushroom body"

Tianqi Wei [1, 2], Qinghai Guo[3], Barbara Webb[1]*

1 Institute of Perception, Action, and Behaviour, School of Informatics, University of Edinburgh, Edinburgh, United Kingdom
2 School of Artificial Intelligence, Sun Yat-sen University, Zhuhai, Guangdong, China
3 Huawei Technologies Co., Ltd., Shenzhen, Guangdong, China

* B.Webb@ed.ac.uk

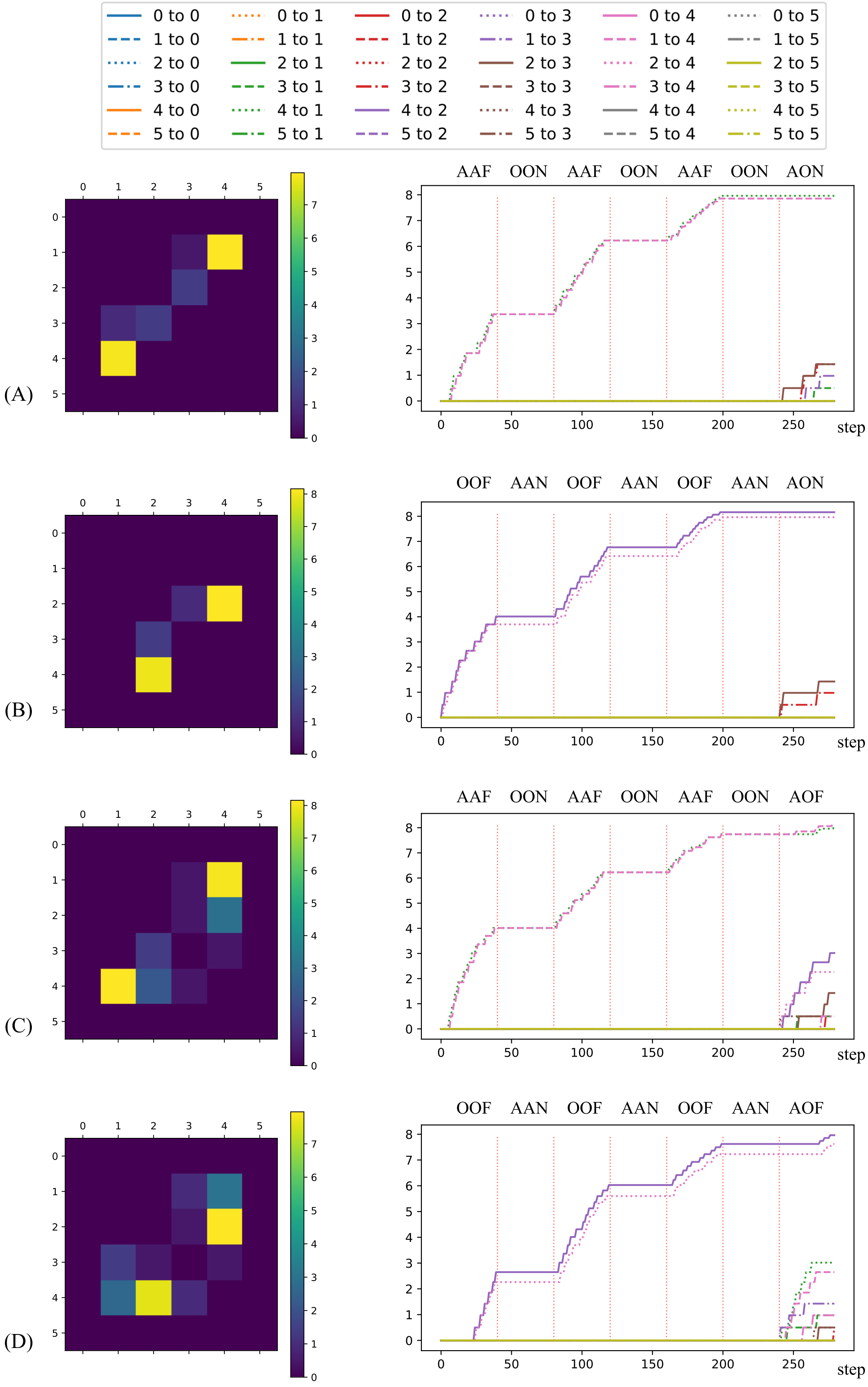

Fig S1. The final synaptic strengths between state nodes and changes during learning of four maggots in four different training protocols. (A) trained with 'AM+/OCT', tested with 'AON', (B) trained with 'AM/OCT+', tested with 'AON', (C) trained with 'AM+/OCT', tested with 'AOF', (D) trained with 'AM/OCT+', tested with 'AOF'. Left column: the final synaptic strengths. Right column: the change of synaptic strengths. Each line is one connection between two states, colour coded as in the legend. The red vertical lines marks the change of Petri dish.
