# Supplementary material for: Learning with sparse reward in a gap junction network inspired by the insect mushroom body
Source: PLoS Comput Biol. 2024 May 23;20(5):e1012086. doi: 10.1371/journal.pcbi.1012086 (PMC11152299; doi:10.1371/journal.pcbi.1012086)
Supplement: S2 Fig — (A) trained with ‘AM+/OCT’, tested with ‘AON’, (B) Trained with ‘AM/OCT+’, tested with ‘AON’, (C) Trained with ‘AM+/OCT’, tested with ‘AOF’, (D) Trained with ‘AM/OCT+’, tested with ‘AOF’. Each line is one connection from edge to action node, colour coded as in the legend. The red vertical lines marks the change of Petri dish. Weights that did not change are omitted. (PDF) [file pcbi.1012086.s002.pdf]

S2 Fig for "Learning with sparse reward in a gap junction network inspired by the insect mushroom body"

Tianqi Wei [1, 2], Qinghai Guo[3], Barbara Webb[1]*

1 Institute of Perception, Action, and Behaviour, School of Informatics, University of Edinburgh, Edinburgh, United Kingdom
2 School of Artificial Intelligence, Sun Yat-sen University, Zhuhai, Guangdong, China
3 Huawei Technologies Co., Ltd., Shenzhen, Guangdong, China

* B.Webb@ed.ac.uk

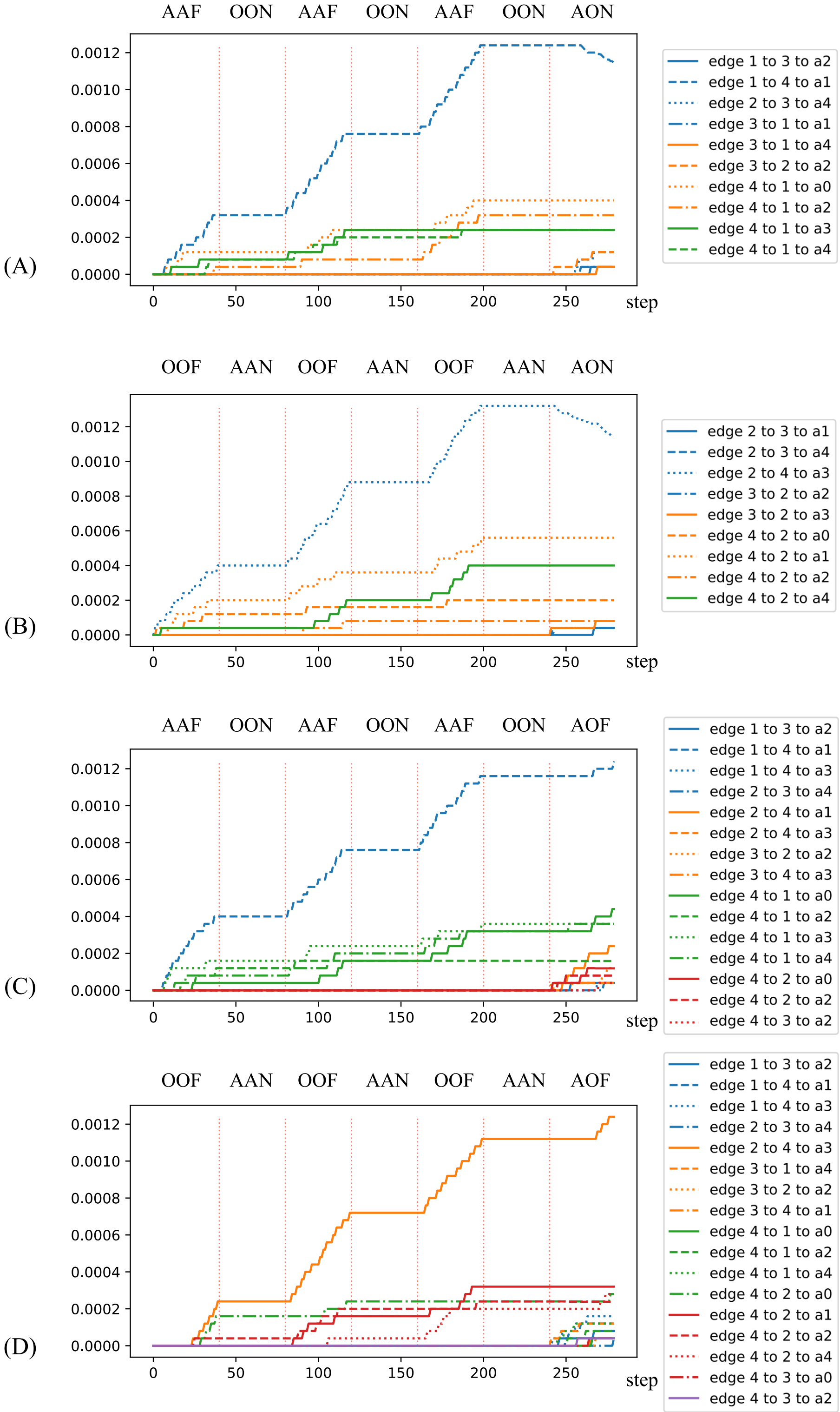

Fig S1. The change of synapse from edges to action nodes during learning of four maggots in four different training protocols. (A) trained with 'AM+/OCT', tested with 'AON', (B) Trained with 'AM/OCT+', tested with 'AON', (C) Trained with 'AM+/OCT', tested with 'AOF', (D) Trained with 'AM/OCT+', tested with 'AOF'. Each line is one connection from edge to action node, colour coded as in the legend. The red vertical lines marks the change of Petri dish. Weights that did not change are omitted.
