# Supplementary material for: Learning with sparse reward in a gap junction network inspired by the insect mushroom body
Source: PLoS Comput Biol. 2024 May 23;20(5):e1012086. doi: 10.1371/journal.pcbi.1012086 (PMC11152299; doi:10.1371/journal.pcbi.1012086)
Supplement: S1 Text — (PDF) [file pcbi.1012086.s003.pdf]

S1 Text for "Learning with sparse reward in a gap junction network inspired by the insect mushroom body"

Tianqi Wei [1, 2], Qinghai Guo[3], Barbara Webb[1*]

1 Institute of Perception, Action, and Behaviour, School of Informatics, University of Edinburgh, Edinburgh, United Kingdom
2 School of Artificial Intelligence, Sun Yat-sen University, Zhuhai, Guangdong, China
3 Huawei Technologies Co., Ltd., Shenzhen, Guangdong, China

* B.Webb@ed.ac.uk

Nodal analysis

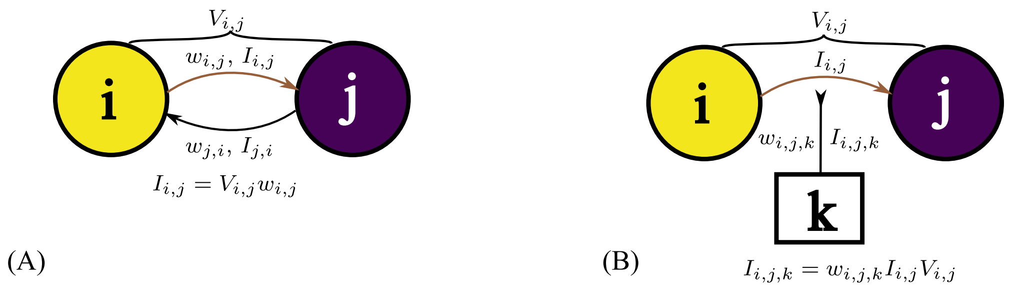

Fig 1. Circuit Analysis for a circuit with bidirectional resistors.

The original nodal analysis is for bidirectional components, such as resistors, as shown in Fig 1. The nodal analysis assumes that the currents flowing from a node to all other nodes the node connects to have a sum zero. Thus, for node 1,

$$w_{12}(\mathrm{v}_1 - \mathrm{v}_2) + w_{13}(\mathrm{v}_1 - \mathrm{v}_3) + \mathrm{i}_{\mathrm{V}_{12}} = 0 \tag{1}$$

Similarly, for node 2 and node 3,

$$w_{12}(\mathrm{v}_2 - \mathrm{v}_1) + w_{23}(\mathrm{v}_2 - \mathrm{v}_3) - \mathrm{i}_{\mathrm{V}_{12}} = 0 \tag{2}$$

$$w_{13}(\mathrm{v}_3 - \mathrm{v}_1) + w_{23}(\mathrm{v}_3 - \mathrm{v}_2) = 0 \tag{3}$$

And apply the potential conservation, assuming v_2 are know:

$$\mathrm{v}_1 = \mathrm{V}_2 + \mathrm{V}_{12} \tag{4}$$

$$\mathrm{v}_2 = \mathrm{V}_2 \tag{5}$$

$$\begin{bmatrix} w_{12}+w_{13} & -w_{12} & -w_{13} & 1 & 0 \\ -w_{12} & w_{12}+w_{23} & -w_{23} & 0 & 1 \\ -w_{13} & -w_{23} & w_{13}+w_{23} & 0 & 0 \\ 1 & 0 & 0 & 0 & 0 \\ 0 & 1 & 0 & 0 & 0 \end{bmatrix} \begin{bmatrix} \mathrm{v}_1 \\ \mathrm{v}_2 \\ \mathrm{v}_3 \\ \mathrm{i}_{\mathrm{V}_{12}} \\ -\mathrm{i}_{\mathrm{V}_{12}} \end{bmatrix} = \begin{bmatrix} 0 \\ 0 \\ 0 \\ \mathrm{V}_2 + \mathrm{V}_{12} \\ \mathrm{V}_2 \end{bmatrix} \tag{6}$$

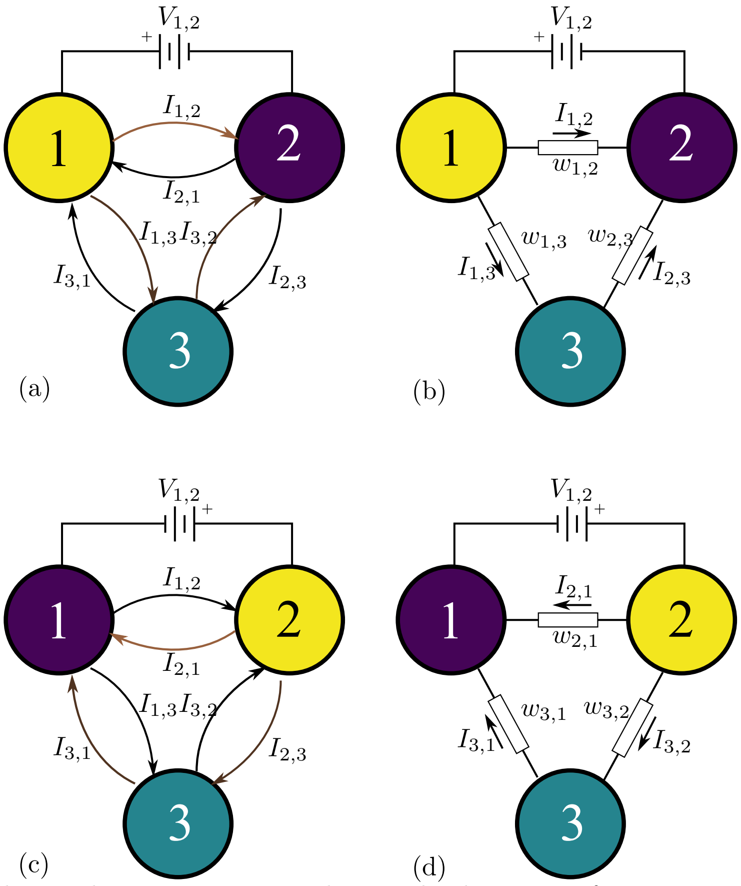

Fig 2. Replacing the resistances according to the directions of currents in circuit analysis

The matrix on the left side is called nodal admittance matrix. It does not have full rank. To solve the equation, one of the nodes can be assumed to be ground, that is, has a potential 0. Thus, the corresponding row and column and be removed.

$$\begin{bmatrix} w_{12}+w_{13} & -w_{13} & 1 & 0 \\ -w_{13} & w_{13}+w_{23} & 0 & 0 \\ 1 & 0 & 0 & 0 \\ 0 & 0 & 0 & 0 \end{bmatrix} \begin{bmatrix} \mathrm{v}_1 \\ \mathrm{v}_3 \\ \mathrm{i}_{\mathrm{V}_{12}} \\ -\mathrm{i}_{\mathrm{V}_{12}} \end{bmatrix} = \begin{bmatrix} 0 \\ 0 \\ \mathrm{V}_{12} \\ 0 \end{bmatrix} \tag{7}$$

Then remove the column and row filled with zeros:

$$\begin{bmatrix} w_{12}+w_{13} & -w_{13} & 1 \\ -w_{13} & w_{13}+w_{23} & 0 \\ 1 & 0 & 0 \end{bmatrix} \begin{bmatrix} \mathrm{v}_1 \\ \mathrm{v}_3 \\ \mathrm{i}_{\mathrm{V}_{12}} \end{bmatrix} = \begin{bmatrix} 0 \\ 0 \\ \mathrm{V}_{12} \end{bmatrix} \tag{8}$$

The above equations can be solved with standard linear algebra solvers, such as torch.linalg.solve() provided by PyTorch. After the potentials are solved, the ground potential can be shifted to a given potential, so as the other potentials.

When applying the nodal analysis to the state network, we take care that the connection in the state network is unidirectional, as shown in Fig 2A and 2B. In this case, the nodal admittance matrix should be updated according to the potentials of the nodes to use the resistance in the direction of the potential gradient (Fig 2B and 2D. Because the circuit in our model has asymmetric connections, the solving process need

several iterations before converging. In each iteration, the potential in the last step is replaced with the new solved potential, the directions of the currents are updated so the corresponding weights are chosen for new equations, and the new equations are solved again.
