# Supplementary material for: Learning with sparse reward in a gap junction network inspired by the insect mushroom body
Source: PLoS Comput Biol. 2024 May 23;20(5):e1012086. doi: 10.1371/journal.pcbi.1012086 (PMC11152299; doi:10.1371/journal.pcbi.1012086)
Supplement: Attachment — Submitted filename: review_comments.pdf [file pcbi.1012086.s004.pdf]

Wei et al. present a resistive network model for application to multi-state tasks such as navigating toward a target location. Nodes in the network represent states, while the strength of connections between nodes represent a preference for state transitions. Weights are learnable such that previously taken state transitions are more likely to be exploited in the future. By grounding a target state node, or learning a current sink at that node, currents can flow through the network, whereby larger currents result in preferences for trajectories through states towards the target state. The model is shown to outperform some standard reinforcement learning algorithms. The paper is mostly easy to follow, however it is lacking in numerous details that would prevent the reader from being able to replicate the work, and make it difficult to interpret some of the results. Wei et al. draw comparisons between features of the model and features of the insect mushroom body, but state that the model is too abstracted from the biology to make specific claims about mushroom body function. As such, in its current form, the motivation for the model, and its applicability to further our understanding of biology, are not very clear, and more methodological details are needed before the reader can interpret the results with confidence. I have outlined in the Major Comments section where greater clarity is needed, and have made several minor suggestions under Minor Comments.

Major Comments

1) To me, the motivation for the work is confused, and the conclusions are difficult to discern, insofar as what novel ideas are being presented. The authors state that current models of mushroom body (MB) function do not address the complex behaviours exhibited by insects. This is true, but that was not their goal, nor does it follow that they cannot be applied to more complex behaviours. The model is inspired by the MB, but is abstracted to the extent that it is difficult to apply results from the model to make predictions about mushroom body function. The model is also clearly inspired by resistive grids, and its relationship to the MB appears more aesthetic, rather than functional in nature. The model is then presented as a resistive grid, without further reference to the MB, and is applied to tasks with no attempt to map those tasks to natural insect behaviour, even though it doesn't seem unreasonable to do so. Novelty therefore is better judged by the comparison of this model with the state of the art in resistive grids. As resistive grids are not in my domain of expertise, I cannot comment on that, but it seems that perhaps unidirectional resistors and experience-dependent resistance may be novel ideas. If so, I think the authors could put greater emphasis on these features, drawing comparisons between their novel model and versions of the model that do not have these novel features. These ideas may well be bio-inspired, as unidirectional gap junctions occur in other species, and I think this would garner interest from a wider audience than saying that they are insect-inspired. I do appreciate that the authors are faced with the tricky task of crossing multiple disciplines and presenting their work to a wide audience. This is not a problem in itself, but the authors would do well to make additional efforts to guide readers across these disciplines, emphasising what is novel, what is standard, and offering more explanations for technical terms. It appears as though the novelty here has more to do with resistive grids than with MB function, and as such, the authors may find a more suitable audience in an engineering/machine learning journal. If the authors wish to target a biologically minded audience, then greater effort is needed to show some biological plausibility in their model, even if it is a generic neural network instead of the MB specifically. Demonstrating that a sparse, distributed state representation does indeed work well for multi-state tasks, rather than having one active node per state, would be a good step in that direction.

2) Much more detail is needed in the methods about the workings of the model and the analyses of results. Methodological details need to be of sufficient detail such that someone can replicate your work without having to resort to the code you provide. More detail is also needed in the figures and their captions. Moreover, figure captions would benefit from greater consistency. E.g. some figures have panels labelled by letters, but those letters aren't referenced in the caption. Some graphs have

unlabelled axes, and other graphs would benefit greatly from legends. Some captions are provided in both the figure panels as well as the dedicated caption text, which is redundant.

3) Line 267: “The subgraph of the states with a passenger in the taxi connects to the other three subgraphs by the action of picking up or dropping off the passenger (Figure 5 (middle))”.
Having only four subgraphs is concerning if there are indeed 5 passenger locations, as stated in lines 234-236. Or was this first mention of 5 passenger locations (and thus the total number of states equalling 500) a mistake. As it stands, this is a major discrepancy.

Minor Comments

1) Abstract: “a problem that remains a challenge for conventional reinforcement learning algorithms”.
Perhaps mention the distal reward problem, as it is often called.

2) Author summary: “ we present a new algorithm for learning in situations where reward is only rarely encountered”.
As a summary for lay people, an explanation as to why this is a problem would help.

3) Author summary: “Input nodes in our network”.
This is perhaps too technical terminology for a non-scientific audience.

4) Author summary: “When a particular goal is chosen or associated with reward”.
The notion of a goal here is a bit vague for a non-scientific audience. Perhaps a brief, concrete example (navigating toward a target seems appropriate) would help.

5) Line 23: “One class of solution to this problem is ”.
References are needed. Are they meant to be 29-31?

6) Line 36: “The KCs extend their axons in parallel through the lobes of the MB, which are innervated by mushroom body output neurons (MBONs) and dopaminergic neurons (DANs). There are multiple compartments in each of the lobes, and to a first approximation, each compartment has one MBON, and one type of DAN.”
References needed, e.g. a recent review paper.
7) Line 42: “Each DAN gets inputs from other brain regions, in particular those that process reinforcing sensory signals, and produces dopamine which modulates the connection between the KCs and the MBON in its specific compartment. ”
References needed, e.g.

- Cohn, R., Morantte, I. & Ruta, V. Coordinated and compartmentalized neuromodulation shapes sensory processing in drosophila. Cell 163, 1742–1755 (2015).
- Hige, T. et al. Heterosynaptic plasticity underlies aversive olfactory learning in drosophila. Neuron 88, 985–998 (2015).
- Owald, D. et al. Activity of defined mushroom body output neurons underlies learned olfactory behavior in drosophila. Neuron 86, 417–427 (2015).
- Schwaerzel, M. et al. Dopamine and octopamine differentiate between aversive and appetitive olfactory memories in drosophila. J. Neurosci. 23, 10495–10502 (2003).
- Mao, Z. & Davis, R. L. Eight different types of dopaminergic neurons innervate the Drosophila mushroom body neuropil : anatomical and physiological heterogeneity. Front. Neural Circuits 3, 1–17 (2009).
- Burke, C. J. et al. Layered reward signalling through octopamine and dopamine in drosophila. Nature 492, 433–437 (2012).

- Liu, C. et al. A subset of dopamine neurons signals reward for odour memory in drosophila. Nature 488, 512–516 (2012).
- Aso, Y. et al. Mushroom body output neurons encode valence and guide memory-based action selection in Drosophila. eLife 3, e04580 (2014).
- Perisse, E. et al. Aversive learning and appetitive motivation toggle feedforward inhibition in the drosophila mushroom body. Neuron 90, 1086–1099 (2016).

8) Figure 1:
- Confusing use of 'top' and 'bottom' in the caption, but 'a' and 'b' in the panels.
- The graphs in the bottom are very crowded and difficult to interpret.
- Is each pink circle a KC? If so, then it appears as though there are many fewer KCs than what was reported. If not, then some explanatory details are needed in the caption.
- What do the text descriptors mean? The terminology needs explaining.
- The text descriptors are too small or overlapping and thus difficult to read. Are they needed at all?

9) Figure 2:
- The electrical circuit diagram may be confusing, as it suggests bidirectional connections between nodes, whereas they are modelled as unidirectional. A more accurate illustration would have 2 connections between every pair of nodes, with diodes specifying the direction.

10) Line 137.
Additional detail would help to explain cov() here, as the covariance cannot be computed until the full sequence of state transitions has finished. Therefore, are we to assume that the means of the gammas are computed using a running average?

11) Line 163: What is meant by sufficient? Sufficient for what?

12) Line 165: What is this constant value?

13) Line 171: “When the target changes, a new target state is set by setting corresponding conductance to ground a large value,”
Revise grammar

14) Line 173: “In this case there is no direct reward function as it assumes the desired state is known, rather than discovered from experience.”
I think it would help if this sentence were swapped with the previous one, to provide better context for the previous sentence.

15) Eq. 7: Given the target audience, I think it would help here if it were stated explicitly how this model departs from plausible biology, insofar as non-local interactions are needed to determine which currents contribute to actions from state nodes, depending on whether or not a state node has the largest potential.

16) Line 196: How does gamma_k enter this equation? Is there a corresponding k_{i,j,k}, and if so, how is it computed?

17) Line 211: “then that”.
Is this meant to read "than the"

18) Line 223: “Because these recent models are based on the proposal that the MB performs temporal difference learning, we used Q-learning as a baseline for comparison.”

Although [24] and [27] do implement a delta-rule for learning, they do not utilise temporal difference learning, nor do they propose that the MB implements TD learning. [42] does not include any computational modelling, so perhaps another citation was meant here?

19) Line 234: “More specifically, the taxi can be in one of five (5) columns, and five (5) rows, have one of four (4) destinations, and there are five (4 + 1) passenger locations, where the extra 1 is when the passenger is in the car, hence in total, 5 × 5 × 4 × 5 = 500 states.”
Is there another way of describing this? It took a few attempts to parse.
An example that I came up with goes:
“There are three features to the state space, 1) the location of the car (with 5x5 = 25 dimensions), 2) the location of the destination (with 4 dimensions), 3) the location of the passenger (either at a pickup location or in the taxi, contributing 4+1 = 5 dimensions). Hence, in total, there are 5x5x4x5=500 states.”
I think it would also be useful to describe how transitions between states are constrained. For example, the passenger-location feature can be changed only when the taxi is at a particular location.

20) Line 254: “we first train the model using only the state of the taxi location”.
Perhaps I don't understand what is meant here, but in Figure 4c, it looks like you are also using the destination as a state, otherwise it wouldn't learn how to reach state 20.

21) Line 259: “It has the same topology as the topology of the grid map.”
It will be helpful to those with less of a mathematical background to provide a more intuitive explanation. As an example:
"The model learns strong gap junction weights between nodes that correspond to possible state transitions in the simulated environment, and weak gap junction weights elsewhere."
I am also sceptical that the topology is indeed the same. Do weights corresponding to impossible state transitions actually get pushed to zero, or are they just very small?

22) Figure 4: It's not necessary, but I think panel C would benefit from an alternative plotting method for the arrows. I know Matlab has graph plotting tools that produce curved arrows so that they overlap less. I think one of your later figures, Fig. 11B, achieves that as well. They can be given different thicknesses, colours, start and end locations etc. There's probably something similar in python, too.
More details are needed in the figure caption, especially for panels A and C.

23) Line 261: “we fed the full state sequence to the model”.
What is a full state sequence? Are state transitions enforced in this case, with no exploration?

24) Line 264: “the passenger has four possible locations: three on the map and one in the taxi.”
Is this meant to be 5 possible locations, 4 pickup locations and one in the taxi, as stated previously?

25) Line 267: “The subgraph of the states with a passenger in the taxi connects to the other three subgraphs by the action of picking up or dropping off the passenger (Figure 5 (middle))”.
Having only four subgraphs is concerning if there are indeed 5 passenger locations. Or, the first mention of 5 passenger locations (and thus the total number of states) was just a simple typo.

26) Figure 5: Much more description is needed in the caption.

27) Line 276: “This performance is comparable with the original results for this task presented in the work by Dietterich [43]”

It is difficult to tell whether the performances are comparable when the reward structure used in this work and in [43] are different. A more detailed explanation of the reward structures are needed. We don't yet know how much reward the taxi gets for completing an episode in the dynamic routing model used here, nor how much reward is given in [43] for completion and per step.

28) Line 279: “Figure 7 shows our model achieved a better performance than the optimal policy Flat Q, and converged at a speed similar to MAXQ Abstract+Greedy and MAXQ + Abstract,”
This is the first time these different variants of Q learning have been mentioned, but they have not been defined or explained anywhere.

29) Figure 6: The performance ranges over several orders of magnitude, and there appear to be two distinct regimes: early (up to episode 100) and late(after episode 250) learning. A log scale, or axes using two different scales separated by a break, would greatly help in seeing the important details in these graphs.

30) Figure 6: I don't understand how the reward per step, in panel B, was computed, if episodes were of variable length.

31) Figure 6: If the reward obtained per episode is always the same, as the taxi must complete the task for an episode to end, then simply plotting number of steps is a more meaningful measure of performance here.

32) Figure 7: “primitive actions” needs to be defined.

33) Figure 8) Why does the model perform so well at the beginning of training?

34) Section 3.2: Grammar in this section needs reviewing.

35) Paragraph starting on Line 289: There are two things being addressed here: the topology of the environment, and the task structure. It comes across as though the Voronoi world enables both a more complex topology *and* a more complex task structure (the possibility to place passengers/destinations at any location). However, the Voronoi world only helps with the topology. A more complex task structure can be achieved in the grid world. Furthermore, an additional sentence stating that the Voronoi world allows for a different number of actions at each state, thus making it more realistic, would also help to provide some intuition about how the environment’s topology affects the task.

36) Line 300: “locations form the Delaunay triangulation of the locations (Figure 10a).”
This is technical language that needs defining/explaining. Some intuition would help here, too, e.g. "if the locations are environment states that are cast as nodes of a graph, Delauny triangulation defines the edges between those nodes that constitute the set of possible state transitions.

37) Line 303: “Some walls are removed by a deep first search algorithm and then a random ratio, thus the corresponding edges of the Delaunay triangulation become paths of the maze.”
Unclear and undefined terminology here. This process needs to be described in the Methods.

38) Line 308: “ Poisson disk sampling [44], which can generate sample locations that avoid small distances between neighbour locations”
Does this mean there is a minimum allowable distance between the target and the agent's start location?

39) Figure 11: A legend is needed to explain weight colours in panel B. What method has been used to remove connections from the learned graph?

40) Line 35: Figure 12 should read Figure 11.

41) Line 340: “Again, we applied Q-learning to the task with a similar configuration, that is, each episode has a large step limit (10000), and rewards are only provided to Q-learning when the target is achieved.”
What about the other Q-learning algorithms that were tested in the previous task?

42) Line 342: “Under this configuration, Q-learning cannot converge, as shown in Figure 13a and 13b, the episode reward oscillates around -2000, and the number of episode steps frequently reaches the limit.”
It has converged if the mean reward per episode has reached a steady state. It's just that the performance is not as good as the dynamic routing model.

43) Line 346: “to examine whether the model can learn with more than one reward source.”
It’s not obvious to me whether this is supposed to be harder or easier to solve. Perhaps the current between the current location and any one target is affected by the presence of other targets, but there are now twice as many opportunities to discover the target by chance.

44) Line 349: “Our model is also able to learn this task quickly, reaching -10 in 214 episodes and converging around 0.”
More details about the reward are needed to make sense of this.

45) Results section: It would be nice to see how the dynamic routing model compares to TD learning, especially given the comparison with TD learning in the Discussion section.

46) Line 371: “ additional models for exploration or computing rewards, which results in more computation and training costs.”
I thought the dynamic routing model also introduces additional models for exploration, treating weights as probabilities and then probabilistically selecting a state transition. More details are needed here to explain why the other models are more computationally demanding.

47) Line 391: “This reduces the size of the transition matrix but also enables generalisation to learning in conditions where the actions of the agent are not the sole determinant of state changes, e.g., learning from observation”
This is confusing to me. Learning from observation hasn't been addressed in the results, or did I miss something?

48) Line 418: “And our model also in principle allows the connection of any two states, not only predefined neighbours, with the connections generated by learning”
Is the learning of state transitions via modulation of the weights/resistances novel? It would be good to state this explicitly, if so. Or has this also been done in the cited works [53-63]?

49) Line 484: “In such a way, our model can be used as a critic in an actor-critic model”
This is an interesting idea, and it would be great to see how the dynamic routing model compares to an actor critic model that uses TD learning. However, in an actor-critic model using TD learning, the value function at adjacent states initially increases, as reward information is propagated along the trajectory taken, and then reduces as state values become more accurate. In the dyanmic routing model, I think the voltage drops would only increase. As such, voltages would implicitly encode a cost per step, which would need to be modelled explicitly in an actor-critic model.
