# Supplementary material for: Learning with sparse reward in a gap junction network inspired by the insect mushroom body
Source: PLoS Comput Biol. 2024 May 23;20(5):e1012086. doi: 10.1371/journal.pcbi.1012086 (PMC11152299; doi:10.1371/journal.pcbi.1012086)
Supplement: Attachment — Submitted filename: Dynamic routing - reply to reviewers.pdf [file pcbi.1012086.s005.pdf]

Learning with sparse reward in a network inspired by the insect mushroom body – response to reviewers

We thank the reviewers for their valuable comments and here provide an explicit response to each comment (shown in red italics, and with relevant line numbers in the revised submission indicated). Note we have also revised the structure of the paper as requested by the editor to fit PLoS CB structure, i.e., moving methods to the end, and hence including a briefer overview of the methods at the start of the results section.

Reviewer #1: In their manuscript "Learning with sparse reward in a network inspired by the insect mushroom body", Wei and co-authors describe computational work in which they investigate a learning algorithm in the area of model-based reinforcement learning. The algorithm utilises a resistor network with asymmetric resistances that are updated according to a local plasticity rule to eventually be a model of the state-space of an RL problem. Activation of nodes of the network represents states of an RL task and transistions (connections between states) are associated with "actions" - presumably the activation of the equivalent of MBONs. The algorithm is tested on the taxiv3 OpenAI task and a more complex task of similar nature developed by the authors.

While the paper is easy to follow in broad strokes, the description of the algorithmic details is hard to understand or contains errors. This makes it very difficult to assess the merits of the work.
For instance, I am unable to parse equation (2). What is the covariance of \Delta \gamma_i, \Delta \gamma_j if \Delta \gamma_i and \Delta \gamma_j are just the changes of the currents injected into state nodes i and j from time step t to t+1 of a discrete-time simulation?
In equation (6), what is the role of the argmax functions for \hat{i} and \hat{j} and how does this relate to equation (7)? In this context, what does "for a larger circuit" mean?
It is never explained - as far as I can see - what the actions actually do in terms of the simulation of the agent/ learning algorithm. There are some confusing references to that the system may take a state that the previous action might not lead to etc which are not clear. It is also not explained what is meant by the system "taking a state" though, reading between the lines, it appears to have something to do with the maximum voltage taken in the network of state nodes.
Another one, equation 8 contains k_{w_{i,j}} but the text below talks about gamma_k. And what is the intuition between the powers 1/2 in the learning rules?

In order to be understood, these model description issues need to be resolved.
One good approach to take in this respect would be to explain one variant of the model fully and in detail; then describe the extensions or simplifications in other variants - not mixing descriptions of all variants together at each level.

We have followed the reviewer's suggestion and described explicitly the discrete state and time variant of the model that is used for the presented results, with brief comments as to how it could be extended or generalised (Methods, lines 575-728). We hope this makes it clearer. Note this revision means some of the specific terms mentioned above are no longer included in the presented equations.

Besides the lacking clarity in the algorithm, there are a couple of other concerns.
First, the learning results are compared to Q learning in which the penalty term (small negative reinforcement) during the trial has been removed. This breaks the ability of Q-learning to accomplish

the task. The authors argue their algorithm does not require the negative reinforcements or trial resets and is hence superior. This doesn't, however, take into account that the new algorithm builds a model during an exploration phase whereas Q learning does not do such a thing. Generally, it is not clear how costly the exploration phase is and how that compares to other approaches, especially other model-based RL algorithms.

It is indeed the building of a model that provides the advantage over Q-learning, specifically when there is no continuous penalty term or resets. However, our algorithm does not have a separate 'exploration phase', that is, it does the same kind of exploration as Q-learning (trying to find actions that maximise reward) with the difference being that it stores information about the experienced state transitions during learning, irrespective of whether these result in reward. As such, it is not costly in time and the storage of information in strengthened connections is not computationally costly. Compared to other model-based RL algorithms, the main advantage is that the use of a resistive network allows for efficient recovery of a trajectory from the current state to a rewarded state without search of the model, i.e., this is also not costly, particularly given its potential to be implemented in hardware.

Lastly, the big elephant in the room is whether this is an algorithm inspired by the insect mushroom body or not. The authors toe a fine line of giving a few similarities of the proposed algorithm with known or recently discovered properties of the mushroom body. But then they wisely use language for the model that sets it apart from the terms used for describing the MB. I suppose that nobody can put in doubt that if the authors say that they were inspired by the mushroom body that it was so. However, is it a helpful assignation to call the model MB-inspired? The authors already point out that assuming potentially all-to-all asymmetric KC gap junctions seems like a stretch. However, there is a much more unrealistic element to their algorithm: actions (presumably MBON activations) are associated with the magnitude of the current flowing through an asymmetric gap junction between two Kenyon cells. The authors need to clarify how this could happen in the nervous system or discuss clearly how the algorithm is clearly not nervous-system-like in this respect and hence should not be interpreted as a model of a brain circuit.

Response: We agree with the reviewer that the abstracted form of the model as presented might appear difficult to interpret as representing mushroom body function. We have tried to clarify that one key idea being explored is that a gap junction network could act as a resistive grid, building on evidence that gap junctions can be assymmetric, adaptive and can modulate chemical synapses (evidence for this is has been added to the introduction, lines 76-89). This significantly expands the potential role of gap junctions (known to exist in many neural systems) in neural computation. It remains highly speculative to suggest they play this role in the insect MB, but we hope at least to bring the potential role to wider attention through our model. Specifically, we suggest that the postsynaptic influence of a KC on an MBON could be modulated by its (electrical) connection to other KC neurons (lines 520-523 and 659-662).

Finally, regarding interest for the general public, while the idea of bio-inspired (or not) machine learning algorithms is clearly interesting to the general public, I feel that this paper would be too technical to be particularly suitable for public outreach.

We assume this is criteria included in the review template but not a requirement for publication?

Reviewer #2: This is an interesting manuscript that proposes a unique neural circuit model for reinforcement learning. The model is inspired by the insect mushroom body (MB), incorporating some recently found ultrastructural features. While I believe this study to be significant and inspiring for people in the field of both computational and experimental neuroscientists, I have several minor concerns regarding how the model relates to actual biology.

Strengths:
The presence of extensive KC-KC connections was discovered only recently, and there have been only one experimental study (Manoim et al., 2022) that addressed one of their functions. To my knowledge, the present manuscript is the first theoretical study to propose another potential benefit of those connections.

The assumptions used in this study (e.g. plasticity of KC-KC or KC-APL connections) provides a few hypotheses potentially testable by experimental biologists.

The “dynamic routing model” proposed in the manuscript shows superior performance than existing reinforcement learning models at least under some sparse reward conditions. The model also significantly differs from typical MB-based circuit models.

The manuscript is well written and accessible for experimental neuroscientists as well.

We thank the reviewer for these positive comments.

Weaknesses:
Although the model is inspired by the recently discovered features of the insect MB circuits, there are several assumptions that are not biologically plausible or oversimplified. While I fully understand that the authors do not intend to claim their model to be an accurate model of the MB, it may diminish the enthusiasm by biologists. Considering the following points could improve the manuscript in this regard.

One of the whole marks of the MB coding is a sparse coding of a large number of stimuli by KCs. The proposed model assumes that a single KC, or state node, responds to a single “world state”. What would happen to the performance of the model if a given state is represented by a sparse (or dense) population of KCs?

Although using a sparse representation for the complete RL tasks is beyond the scope of the present work, we also include in the discussion a simple demonstration that the principle of current flow from input state to target state still holds when both states are represented by groups of (potentially overlapping) nodes rather than single nodes (lines 530-551 and figure 14).

It is not clear what kind of synaptic transmission and plasticity the authors are assuming to take place at KC-MBON synapses, or “action node”. It would help if they can propose it using biological terms.

As implemented, we assume that the activation of an action node depends on the strength of current flow between the state nodes to which it is connected, and these connections form (are plastic) when the

action causes transitions between states. In biological terms, this would suggest that how much an active KC contributes to MBON activation is modulated by that KC's connectivity to other KCs, for example, that gap junctions between KC axons alters the strength of the electrical signal before it reaches the chemical synapse onto the MBON. We have added some sentences to the model description and discussion to explain this concept (lines 520-523 and 659-662).

In line 221, the authors claim that their tasks are more difficult than the ones previously used in the MB models. I am curious to know how the authors' model with lateral connections between KCs performs in those simpler associative learning tasks. This may be out of the scope of the manuscript, but perhaps the authors could comment on it in Discussion.

We have now included a new task of olfactory associative learning to show that the abstracted model can learn the 'simpler' problem addressed by previous MB models (lines 305-406). This raises some interesting issues for how such classical conditioning paradigms should be mapped to RL which we briefly discuss.

KC-KC chemical connections are currently considered inhibitory (Manoim et al., 2022). The model is perhaps rather inspired by the presence of gap junctions, but it is difficult imagine that gap junctions are as plastic as the model assumes. Even if they are, it is unclear how the excitation in one KC propagates through multiple KCs via such gap junctions. Perhaps, the authors could test what happens if KCs are not connected in an all-to-all fashion but communicate more locally.

We have expanded on the discussion of gap junctions in the introduction (lines 76-89) including evidence that gap junctions are both adaptive and can modulate chemical synapses. This significantly expands the potential role of gap junctions (known to exist in many neural systems) in neural computation. We agree it remains highly speculative to suggest they form a resistive grid representing state-transitions in the MB, but we hope at least to bring the potential role to wider attention through our model. We have added short discussion of how changing from (potential) all-to-all connections might affect the model (lines 525-529) - essentially, it could impose innate constraints on what could be learnt - and this is something we plan to explore in future coupled with the use of sparse (rather than one-hot) coding of the state.

<u>Reviewer #3:</u> Wei et al. present a resistive network model for application to multi-state tasks such as navigating toward a target location. Nodes in the network represent states, while the strength of connections between nodes represent a preference for state transitions. Weights are learnable such that previously taken state transitions are more likely to be exploited in the future. By grounding a target state node, or learning a current sink at that node, currents can flow through the network, whereby larger currents result in preferences for trajectories through states towards the target state. The model is shown to outperform some standard reinforcement learning algorithms. The paper is mostly easy to follow, however it is lacking in numerous details that would prevent the reader from being able to replicate the work, and make it difficult to interpret some of the results. Wei et al. draw comparisons between features of the model and features of the insect mushroom body, but state that the model is too abstracted from the biology to make specific claims about mushroom body function. As such, in its current form, the motivation for the model, and its applicability to further our understanding of biology, are not very clear, and more methodological details are needed before the reader can

interpret the results with confidence. I have outlined in the Major Comments section where greater clarity is needed, and have made several minor suggestions under Minor Comments.

We thank the reviewer for this thorough review.

Major Comments

1) To me, the motivation for the work is confused, and the conclusions are difficult to discern, insofar as what novel ideas are being presented. The authors state that current models of mushroom body (MB) function do not address the complex behaviours exhibited by insects. This is true, but that was not their goal, nor does it follow that they cannot be applied to more complex behaviours. The model is inspired by the MB, but is abstracted to the extent that it is difficult to apply results from the model to make predictions about mushroom body function. The model is also clearly inspired by resistive grids, and its relationship to the MB appears more aesthetic, rather than functional in nature. The model is then presented as a resistive grid, without further reference to the MB, and is applied to tasks with no attempt to map those tasks to natural insect behaviour, even though it doesn't seem unreasonable to do so. Novelty therefore is better judged by the comparison of this model with the state of the art in resistive grids. As resistive grids are not in my domain of expertise, I cannot comment on that, but it seems that perhaps unidirectional resistors and experience-dependent resistance may be novel ideas. If so, I think the authors could put greater emphasis on these features, drawing comparisons between their novel model and versions of the model that do not have these novel features. These ideas may well be bio-inspired, as unidirectional gap junctions occur in other species, and I think this would garner interest from a wider audience than saying that they are insect-inspired. I do appreciate that the authors are faced with the tricky task of crossing multiple disciplines and presenting their work to a wide audience. This is not a problem in itself, but the authors would do well to make additional efforts to guide readers across these disciplines, emphasising what is novel, what is standard, and offering more explanations for technical terms. It appears as though the novelty here has more to do with resistive grids than with MB function, and as such, the authors may find a more suitable audience in an engineering/machine learning journal.

Response: We agree with the reviewer that the model as presented does not make explicit predictions about mushroom body function, given the abstracted nature of the model and the evaluation against standard RL tasks rather than insect learning paradigms. We have now included a new task of olfactory associative learning (lines 305-406) to show that the abstracted model can learn the 'simpler' problem addressed by previous MB models. But more importantly, we have clarified that one key idea being explored is that a gap junction network could act as a resistive grid, building on evidence that gap junctions are both adaptive and can modulate chemical synapses (added to introduction, lines 76-89). This significantly expands the potential role of gap junctions (known to exist in many neural systems) in neural computation. It remains highly speculative to suggest they play this role in the insect MB, but we hope at least to bring the potential role to wider attention through our model.

If the authors wish to target a biologically minded audience, then greater effort is needed to show some biological plausibility in their model, even if it is a generic neural network instead of the MB specifically. Demonstrating that a sparse, distributed state representation does indeed work well for multi-state tasks, rather than having one active node per state, would be a good step in that direction.

Response: We have expanded on the discussion of gap junctions in the introduction (lines 76-89) which we hope increases the support for biological plausibility. Although using a sparse representation for the complete RL tasks is beyond the scope of the present work, we also include in the discussion (lines 530-551 and figure 14) a simple demonstration that the principle of current flow from input state to target state still holds when both states are represented by groups of (potentially overlapping) nodes rather than single nodes.

2) Much more detail is needed in the methods about the workings of the model and the analyses of results. Methodological details need to be of sufficient detail such that someone can replicate your work without having to resort to the code you provide. More detail is also needed in the figures and their captions. Moreover, figure captions would benefit from greater consistency. E.g. some figures have panels labelled by letters, but those letters aren't referenced in the caption. Some graphs have unlabelled axes, and other graphs would benefit greatly from legends. Some captions are provided in both the figure panels as well as the dedicated caption text, which is redundant.

We have revised the methods (as also suggested by reviewer 1) and the figures and captions (following the detailed minor comments below) and hope that the approach is now clear.

3) Line 267: "The subgraph of the states with a passenger in the taxi connects to the other three subgraphs by the action of picking up or dropping off the passenger (Figure 5 (middle))". Having only four subgraphs is concerning if there are indeed 5 passenger locations, as stated in lines 234-236. Or was this first mention of 5 passenger locations (and thus the total number of states equalling 500) a mistake. As it stands, this is a major discrepancy.

There are in principle 500 discrete states but only 400 that can be reached during an episode, because when the passenger location is the same as their destination, the episode terminates. We have added some sentences to make this clear (lines 168-170)

Minor Comments

1) Abstract: "a problem that remains a challenge for conventional reinforcement learning algorithms". Perhaps mention the distal reward problem, as it is often called.

We have made this change as suggested (second sentence of abstract).

2) Author summary: " we present a new algorithm for learning in situations where reward is only rarely encountered". As a summary for lay people, an explanation as to why this is a problem would help.

3) Author summary: "Input nodes in our network". This is perhaps too technical terminology for a non-scientific audience.

4) Author summary: "When a particular goal is chosen or associated with reward". The notion of a goal here is a bit vague for a non-scientific audience. Perhaps a brief, concrete example (navigating toward a target seems appropriate) would help.

We have updated the author summary following suggestions 2-4 (lines 1-12)

5) Line 23: "One class of solution to this problem is ". References are needed. Are they meant to be 29-31?

Reference added (line 36)

6) Line 36: “The KCs extend their axons in parallel through the lobes of the MB, which are innervated by mushroom body output neurons (MBONs) and dopaminergic neurons (DANs). There are multiple compartments in each of the lobes, and to a first approximation, each compartment has one MBON, and one type of DAN.”

References needed, e.g. a recent review paper.

Reference added (line 53).

7) Line 42: “Each DAN gets inputs from other brain regions, in particular those that process reinforcing sensory signals, and produces dopamine which modulates the connection between the KCs and the MBON in its specific compartment. ”

References needed, e.g.

• Cohn, R., Morantte, I. & Ruta, V. Coordinated and compartmentalized neuromodulation shapes sensory processing in drosophila. Cell 163, 1742–1755 (2015).

• Hige, T. et al. Heterosynaptic plasticity underlies aversive olfactory learning in drosophila. Neuron 88, 985–998 (2015).

• Owald, D. et al. Activity of defined mushroom body output neurons underlies learned olfactory behavior in drosophila. Neuron 86, 417–427 (2015).

• Schwaerzel, M. et al. Dopamine and octopamine differentiate between aversive anappetitive olfactory memories in drosophila. J. Neurosci. 23, 10495–10502 (2003).

• Mao, Z. & Davis, R. L. Eight different types of dopaminergic neurons innervate the Drosophila mushroom body neuropil : anatomical and physiological heterogeneity. Front. Neural Circuits 3, 1–17 (2009).

• Burke, C. J. et al. Layered reward signalling through octopamine and dopamine in drosophila. Nature 492, 433–437 (2012).

Liu, C. et al. A subset of dopamine neurons signals reward for odour memory in drosophila. Nature 488, 512–516 (2012).

• Aso, Y. et al. Mushroom body output neurons encode valence and guide memory-based action selection in Drosophila. eLife 3, e04580 (2014).

• Perisse, E. et al. Aversive learning and appetitive motivation toggle feedforward inhibition in the drosophila mushroom body. Neuron 90, 1086–1099 (2016).

A selection of these references has been added (line58).

8) Figure 1:

- Confusing use of 'top' and 'bottom' in the caption, but 'a' and 'b' in the panels.

Fixed.

- The graphs in the bottom are very crowded and difficult to interpret.

- Is each pink circle a KC? If so, then it appears as though there are many fewer KCs than what was reported. If not, then some explanatory details are needed in the caption.

- What do the text descriptors mean? The terminology needs explaining.

- The text descriptors are too small or overlapping and thus difficult to read. Are they needed at all?

We have revised the caption to explain these details.

9) Figure 2:

- The electrical circuit diagram may be confusing, as it suggests bidirectional connections between nodes, whereas they are modelled as unidirectional. A more accurate illustration would have 2 connections between every pair of nodes, with diodes specifying the direction.

We have made the suggested change in figure 2.

10) Line 137. Additional detail would help to explain cov() here, as the covariance cannot be computed until the full sequence of state transitions has finished. Therefore, are we to assume that the means of the gammas are computed using a running average?

We have revised the description of the model to be specific to the simplified (discrete) case implemented in this work (see also response to reviewer 1) which has removed the reference to covariance.

11) Line 163: What is meant by sufficient? Sufficient for what?

'Sufficient' has been removed.

12) Line 165: What is this constant value?

Clarified in text that this value is not critical, provided it is larger than conductance between KCs (lines 676-678).

13) Line 171: "When the target changes, a new target state is set by setting corresponding conductance to ground a large value," Revise grammar

Revised (lines 670-672).

14) Line 173: "In this case there is no direct reward function as it assumes the desired state is known, rather than discovered from experience." I think it would help if this sentence were swapped with the previous one, to provide better context for the previous sentence.

Clarified in text (lines 681-686)

15) Eq. 7: Given the target audience, I think it would help here if it were stated explicitly how this model departs from plausible biology, insofar as non-local interactions are needed to determine which currents contribute to actions from state nodes, depending on whether or not a state node has the largest potential.

We hope the revised explanation (lines 627-656) makes the process clear: the currents contributing to actions are determined by taking the current state node and determining which of the `edges' from this node to connected states has the strongest current (equation 3); so we consider this to be a local interaction. Because an edge could connect to multiple action nodes we include equation 4 to choose between actions; this could be considered to be a mechanism of lateral inhibition between the action nodes.

16) Line 196: How does gamma_k enter this equation? Is there a corresponding k_{i,j,k}, and if so, how is it computed?

This variable is no longer relevant in the revised methods.

17) Line 211: "then that". Is this meant to read "than the"

No longer included.

18) Line 223: "Because these recent models are based on the proposal that the MB performs temporal difference learning, we used Q-learning as a baseline for comparison." Although [24] and [27] do implement a delta-rule for learning, they do not utilise temporal difference learning, nor do they propose that the MB implements TD learning. [42] does not include any computational modelling, so perhaps another citation was meant here?

We have changed this from 'temporal difference learning' to 'prediction error learning' (lines 146-148). Indeed this was an incorrect citation, we have changed it to refer to Springer & Nawrot 2021 (line 144).

19) Line 234: "More specifically, the taxi can be in one of five (5) columns, and five (5) rows, have one of four (4) destinations, and there are five (4 + 1) passenger locations, where the extra 1 is when the passenger is in the car, hence in total, 5 × 5 × 4 × 5 = 500 states." Is there another way of describing this? It took a few attempts to parse.

An example that I came up with goes: "There are three features to the state space, 1) the location of the car (with 5x5 = 25 dimensions), 2) the location of the destination (with 4 dimensions), 3) the location of the passenger (either at a pickup location or in the taxi, contributing 4+1 = 5 dimensions). Hence, in total, there are 5x5x4x5=500 states."

I think it would also be useful to describe how transitions between states are constrained. For example, the passenger-location feature can be changed only when the taxi is at a particular location.

We have updated the description as suggested (lines 160-167).

20) Line 254: "we first train the model using only the state of the taxi location". Perhaps I don't understand what is meant here, but in Figure 4c, it looks like you are also using the destination as a state, otherwise it wouldn't learn how to reach state 20.

We have clarified that this initial training is for the taxi to reach a target location (lines 187-189).

21) Line 259: "It has the same topology as the topology of the grid map." It will be helpful to those with less of a mathematical background to provide a more intuitive explanation. As an example: "The model learns strong gap junction weights between nodes that correspond to possible state transitions in the simulated environment, and weak gap junction weights elsewhere." I am also sceptical that the topology

is indeed the same. Do weights corresponding to impossible state transitions actually get pushed to zero, or are they just very small?

We have modified this description as suggested (lines 193-196).

22) Figure 4: It's not necessary, but I think panel C would benefit from an alternative plotting method for the arrows. I know Matlab has graph plotting tools that produce curved arrows so that they overlap less. I think one of your later figures, Fig. 11B, achieves that as well. They can be given different thicknesses, colours, start and end locations etc. There's probably something similar in python, too.

We appreciate the comment but have decided not to modify the figure; we hope the updated caption is sufficient.

More details are needed in the figure caption, especially for panels A and C.

We have added more detail to the figure caption.

23) Line 261: "we fed the full state sequence to the model". What is a full state sequence? Are state transitions enforced in this case, with no exploration?

We have clarified what is happening in this training with the full state space (lines 197-198).

24) Line 264: "the passenger has four possible locations: three on the map and one in the taxi." Is this meant to be 5 possible locations, 4 pickup locations and one in the taxi, as stated previously?

See above, we have clarified that the fourth location, at their destination, leads to termination of the episode so is not represented (lines 203-205).

25) Line 267: "The subgraph of the states with a passenger in the taxi connects to the other threesubgraphs by the action of picking up or dropping off the passenger (Figure 5 (middle))".Having only four subgraphs is concerning if there are indeed 5 passenger locations. Or, the first mention of 5 passenger locations (and thus the total number of states) was just a simple typo.

Same as response to major comment: There are in principle 500 discrete states but only 400 that can be reached during an episode, because when the passenger location is the same as their destination, the episode terminates. We have added some sentences to make this clear (lines 168-170)

26) Figure 5: Much more description is needed in the caption.

More description added.

27) Line 276: "This performance is comparable with the original results for this task presented in the work by Dietterich [43]" It is difficult to tell whether the performances are comparable when the reward structure used in this work and in [43] are different. A more detailed explanation of the reward structures are needed. We don't yet know how much reward the taxi gets for completing an episode in the dynamic routing model used here, nor how much reward is given in [43] for completion and per step.

We have added a more detailed description of the rewards used in our training and plots (lines 351-355) and in previous implementations of this task (lines 210-214).

28) Line 279: “Figure 7 shows our model achieved a better performance than the optimal policy Flat Q, and converged at a speed similar to MAXQ Abstract+Greedy and MAXQ + Abstract,”

This is the first time these different variants of Q learning have been mentioned, but they have not been defined or explained anywhere.

We have now removed this figure as we have noticed that some other small differences in the task set-up in Dietterich (2000) that make direct comparison inappropriate. We provide a brief verbal comparison, explaining that they use hierarchical RL to obtain faster convergence, and the convergence time in our model is comparable (lines 218-223).

29) Figure 6: The performance ranges over several orders of magnitude, and there appear to be two distinct regimes: early (up to episode 100) and late(after episode 250) learning. A log scale, or axes using two different scales separated by a break, would greatly help in seeing the important details in these graphs.

We tried to use the inset plots to show this, and hope this is sufficient; in particular these illustrate small continuing improvement in the reward.

30) Figure 6: I don't understand how the reward per step, in panel B, was computed, if episodes were of variable length.

Panel B essentially shows the same as panel A, i.e. reward per episode but plotted against steps on the x-axis to illustrate that episodes could contain very different numbers of steps. We have updated the caption to clarify.

31) Figure 6: If the reward obtained per episode is always the same, as the taxi must complete the task for an episode to end, then simply plotting number of steps is a more meaningful measure of performance here.

As we have tried to clarify in the text (lines 210-214) to allow more direct comparison with previous work using the same task, we calculate the episode reward in these plots inclusive of a negative reward per step or for incorrect destinations, although these are not used in the actual training algorithm; hence the reward per episode can vary. Steps per episode is already plotted, in figure 8.

32) Figure 7: “primitive actions” needs to be defined.

We could not determine what this comment refers to, possibly it has gone after the revision?

33) Figure 8) Why does the model perform so well at the beginning of training?

Now figure 7, we have commented on the apparent decrease from a better performance at the start of training although we are unsure why the average is higher at the outset.

34) Section 3.2: Grammar in this section needs reviewing.

Hopefully fixed.

35) Paragraph starting on Line 289: There are two things being addressed here: the topology of the environment, and the task structure. It comes across as though the Voronoi world enables both a more

complex topology and a more complex task structure (the possibility to place passengers/destinations at any location). However, the Voronoi world only helps with the topology. A more complex task structure can be achieved in the grid world. Furthermore, an additional sentence stating that the Voronoi world allows for a different number of actions at each state, thus making it more realistic, would also help to provide some intuition about how the environment's topology affects the task.

We have updated this description as suggested (line 237-247).

36) Line 300: "locations form the Delaunay triangulation of the locations (Figure 10a)." This is technical language that needs defining/explaining. Some intuition would help here, too, e.g."if the locations are environment states that are cast as nodes of a graph, Delauny triangulation defines the edges between those nodes that constitute the set of possible state transitions.

We have rephrased as suggested (lines 257-261).

37) Line 303: "Some walls are removed by a deep first search algorithm and then a random ratio, thus the corresponding edges of the Delaunay triangulation become paths of the maze." Unclear and undefined terminology here. This process needs to be described in the Methods.

We have updated our description of this process (lines 253-256)

38) Line 308: " Poisson disk sampling [44], which can generate sample locations that avoid small distances between neighbour locations" Does this mean there is a minimum allowable distance between the target and the agent's start location?

No, this is referring to all possible locations the agent can occupy on the 2D plane, the yellow dots in the figure. We have modified the text to make this clear (lines 242-247).

39) Figure 11: A legend is needed to explain weight colours in panel B. What method has been used to remove connections from the learned graph?

We have added information in the caption to explain that the darker the colour, the stronger the connection and that connections with weights below a threshold are not shown.

40) Line 335: Figure 12 should read Figure 11.

Fixed (now figure 10)

41) Line 340: "Again, we applied Q-learning to the task with a similar configuration, that is, each episode has a large step limit (10000), and rewards are only provided to Q-learning when the target is achieved."

What about the other Q-learning algorithms that were tested in the previous task?

Note we did not implement the other Q-learning algorithms (for the previous task) but reported previously published results for them for comparison. Here, as it is new task, there are no previously published results.

42) Line 342: "Under this configuration, Q-learning cannot converge, as shown in Figure 13a and 13b, the episode reward oscillates around -2000, and the number of episode steps frequently reaches

the limit.” It has converged if the mean reward per episode has reached a steady state. It's just that the performance is not as good as the dynamic routing model.

We do not think this has reached a steady state.

43) Line 346: “to examine whether the model can learn with more than one reward source.” It’s not obvious to me whether this is supposed to be harder or easier to solve. Perhaps the current between the current location and any one target is affected by the presence of other targets, but there are now twice as many opportunities to discover the target by chance.

Potentially this is an ‘easier’ task but the point of this test was to ensure that the model does not depend on having only one target to create the appropriate current flow, but rather that the flow with several targets is also appropriate for solving the problem.

44) Line 349: “Our model is also able to learn this task quickly, reaching -10 in 214 episodes and converging around 0.” More details about the reward are needed to make sense of this.

Details have been added (lines 287-291).

45) Results section: It would be nice to see how the dynamic routing model compares to TD learning, especially given the comparison with TD learning in the Discussion section.

We discuss multiple alternative RL methods in this section and it is not clear which would be most appropriate as a comparison – possibly the successor representation would be most interesting, but this is beyond the scope of the current revision.

46) Line 371: “ additional models for exploration or computing rewards, which results in more computation and training costs.”

I thought the dynamic routing model also introduces additional models for exploration, treating weights as probabilities and then probabilistically selecting a state transition. More details are needed here to explain why the other models are more computationally demanding.

The simple point being made in this sentence is that these methods are more computationally demanding than standard RL, rather than directly comparing the computation required by these methods with our method; as such we do not see a need to change the statement. We have added a sentence to clarify that the relevant comparison here is whether subgoals need to be explicitly introduced (as in the previous methods described) or can emerge from one mechanism (the currents in our model), lines 432-435.

47) Line 391: “This reduces the size of the transition matrix but also enables generalisation to learning in conditions where the actions of the agent are not the sole determinant of state changes, e.g., learning from observation” This is confusing to me. Learning from observation hasn't been addressed in the results, or did I miss something?

We have clarified that learning from observation is not tested here (lines 453-455) but it seems clear that if the agent experiences state changes that are caused by another agent, it will learn about those changes. Perhaps this should not be called learning from observation as the agent would not observe the actions that led to the change, but learning the state transitions could structure subsequent learning of correct actions.

48) Line 418: “And our model also in principle allows the connection of any two states, not only predefined neighbours, with the connections generated by learning” Is the learning of state transitions via modulation of the weights/resistances novel? It would be good to state this explicitly, if so. Or has this also been done in the cited works [53-63]?

Relative to the previous work on resistive networks for planning it is novel and we have now stated this explicitly (lines 479-482).

49) Line 484: “In such a way, our model can be used as a critic in an actor-critic model” This is an interesting idea, and it would be great to see how the dynamic routing model compares to an actor critic model that uses TD learning. However, in an actor-critic model using TD learning, the value function at adjacent states initially increases, as reward information is propagated along the trajectory taken, and then reduces as state values become more accurate. In the dynamic routing model, I think the voltage drops would only increase. As such, voltages would implicitly encode a time cost per step, which would need to be modelled explicitly in an actor-critic model.

Thankyou for this comment, this is something we plan to look at more closely in future.
