# Supplementary material for: Learning with sparse reward in a gap junction network inspired by the insect mushroom body
Source: PLoS Comput Biol. 2024 May 23;20(5):e1012086. doi: 10.1371/journal.pcbi.1012086 (PMC11152299; doi:10.1371/journal.pcbi.1012086)
Supplement: Attachment — Submitted filename: reviewer_comments.pdf [file pcbi.1012086.s006.pdf]

Overview
The authors have made excellent improvements to the flow and readability of the manuscript. They have introduced a new results section, applying their dynamic routing model to a typical conditioning experiment performed with insects. Unfortunately, this section introduces several new sources of confusion that would benefit from clarification.

Major Comments
Simple associative learning – there are quite a few ambiguities in this application of the model, and as a result I am finding it very difficult to make sense of it. I appreciate that the authors are trying to be concise, but I think some details need to be stated explicitly, and others would benefit from rewording to make the explanations more clear.

1. It's not clear to me why, in the test phase without reinforcement, the maggot keeps selecting the previously reinforced odour, but does so much less when there is reinforcement in the test phase. E.g., take training protocol 1 (training with AM+/OCT and test petri dish AON). During training, state transition state1→state4 is eventually selected with action "toward AM", resulting in that state transition and that action being strengthened. The stronger it becomes, the more likely it is selected, and so it becomes even stronger. Then, in the test phase, I presume the state1→state4 connection has the max power (though this needs to be stated explicitly if so, as it is not obviously the case when state 4 is no longer grounded), and the "toward AM" action continues to be chosen. However, because this action no longer results in the maggot occupying state 4, but rather state 1 again (as there is no reward), the "toward AM" action is weakened, making it less likely to be chosen again. Maybe the action's weight is so strong that this doesn't matter, but after 40 steps I would have thought it would become significantly weaker. Either way, the consequences need to be explained as it's not obvious how the model will evolve (diagrams and figures showing the evolution of action weights and state connection weights etc. would be a great help). Conversely, in protocol 3, when there is reinforcement during the test phase (petri dish AOF), state 4 is grounded (or as before, the state1→state4 weight is large), giving that connection high power, and the action "select AM" results in the maggot occupying state 4, thus further strengthening the action. At least, that is how I understand it.
2. Maybe this is related to point 1 above, but it's not clear to me what happens when the maggot moves to state 4. Is it automatically moved back to state 1 after the reward is received, as it is in the presence of odour AM, after all. Or does it then have to move from state 4 to another state in the next step? If the latter, and it selects "toward AM" again, is the maggot kept in state 4 (thus weakening the action "toward AM" because it stems from connections to anywhere but state 4), or does it move to state 1, allowing the action to be strengthened?
3. If it is indeed possible for the maggot to remain in the same state over consecutive steps, are self-connections therefore incorporated in this paradigm?
4. If the maggot is in state 1 (AM present), there is presumably no odour gradient. Are actions always random in this case, even if there is OCT elsewhere in the petri dish? I.e. is an odour gradient only possible in petri dishes with both odours AND when the maggot is in state 3? Or is the maggot aware of the presence of an odour gradient in AON/AOF/AOG dishes, even when it is not actually in a gradient containing state? Whichever it is, it needs explaining explicitly.
5. Line 337-339 – Take the AM+/OCT training protocol. Am I right in thinking that the agent only receives fructose reward if it is on an AM state, and then approaches the other AM state? As though a maggot has to crawl to the far end of a corridor before it receives a reward. If so, could the sentence starting "When a maggot takes an appetitive action…" be rephrased to make this more explicit.

6. Line 373 – Is the fact that there is no current flow because the high potential due to being in state 4 is countered by the grounded potential due to the presence of reward? This needs a more clear explanation.
7. Table 1 – During testing, for petri dishes with two odours and a reinforcement, it needs to be stated which odour receives the reinforcement. Initially, I presumed it was the same as the odour that received reinforcement during training, but lines 379-381 make me think that perhaps both odours can provide the same reinforcement. Either way, this needs to be made explicit.
8. It's not clear to me when the default state is activated, if ever.

<u>Minor Comments</u>

9. Table 5 – "appetite" and "averse" maybe better replaced with "toward" and "escape", to be consistent with language used previously.
10. Line 646-647 – It's stated that Eq. 6 is equivalent to some expression given in Eq. 8, but I don't think this is so. I think Eq. 6 should always depend on the power consumption. It is the term inside the parenthesis in Eq. 7 that is equivalent to Eq. 8, as that is where the power is cancelled out. Thus, the term inside the parenthesis is equivalent to c_1 * w_ijk / max_k(w_ijk)
11. Line 368 – "state 3 (fructose)" seems incorrect. Fructose is state 4.
12. Line 603 – It is not clear what "Assumption 1" is, but interpreting the rest of the sentence depends on it.
13. Line 657-662 – I think some additional explanation would be helpful here, especially in terms of pointing out the caveats of this interpretation. In particular – and if I understand the mechanisms properly – the biological interpretation that has been given would make more sense if all connections emanating from a particular node contributed to the current received by a particular action, rather than the current being a function of the connection to the node with the highest power consumption only. The approximation, ie. whereby only the connection to the highest power node drove the action, would then make sense if the inverse temperature in the softmax function was very high.
14. Line 157 – it is a bit confusing to say there are 3 dimensions, when each dimension comprises some number of other dimensions.
15. RE minor comment 15 in my previous review – Regarding the selection of a connection to the highest power node, I believe a clear explanation of why this is a local operation is needed. I don't yet see how it is local, as the synaptic connection itself doesn't have local access to the necessary information needed to effectively gate open/closed "second-order" connections to the action nodes. Even though the connections share a common neuron (that of the current state), I don't yet see how information about the potentials at connected nodes is shared between connections. In a standard neural circuit model, it would be unusual for a synapse between neuron A and B to be silenced by a stronger synapse between A and C, apart from when some normalisation is used after a weight update, which itself is a non-local operation, and in any case is not the mechanism at play here.
16. RE minor comment 30 in my previous review – I didn't properly understand the new caption for Fig 5 until I read the authors' response to my comment. I think the caption should be replaced with the authors' response to clearly explain what is going on.
17. Line 258 – It states that edges without walls are the passable edges, but also that every edge crosses a wall, suggesting that, in fact, no edges are passable. I think it might be easier to follow if the construction of the Voronoi world is described in this order: 1) Voronoi walls are constructed, 2) Delauney triangulation constructed, 3) a random selection of Voronoi walls are removed, 4) corresponding Delauney edges are removed, leaving only edges that an agent can follow around the walls.
18. Fig. 8 – Can numbers be added to panel B to aid comparison between A and B?

19. Fig. 9 – there is some spurious information in panel A, “step 14 | Fuel left: 9987 | Rewards: 19”, which doesn’t seem relevant.
