# Supplementary material for: Learning with sparse reward in a gap junction network inspired by the insect mushroom body
Source: PLoS Comput Biol. 2024 May 23;20(5):e1012086. doi: 10.1371/journal.pcbi.1012086 (PMC11152299; doi:10.1371/journal.pcbi.1012086)
Supplement: Attachment — Submitted filename: Response to reviews.pdf [file pcbi.1012086.s007.pdf]

We thank the reviewers for their close attention to our paper, and have tried to respond here to all comments. The reviewers comments are shown as plain text and our responses in *red italics.*

Reviewer #1:

In the last decade, there have been a number of works that used the insect mushroom body as an inspiration for investigating certain neural networks and, increasingly, also for proposing more abstract models of learning. However, after studying the review reports of other reviewers, the response of the authors and the revised manuscript I feel this manuscript is going too far. While the methods are still not presented well (more about this below), it is now becoming clearer how the model works. This highlights that the model is an algorithmic model, inspired by resistor networks. The attempts of linking the main mechanisms of the model to insect mushroom bodies are contrived. For instance, Kenyon cells are spiking neurons which is hard to reconcile with being nodes in a resistor network. What would it mean for a KC to be "pulled down" or "pulled up"? Gap junctions between KCs wouldn't just create a continuous field of current flows like the nodes in the resistor network.

We have revised our introduction (line 13 onward) to clarify that our conception relates to axo-axonal interaction of the KCs in the MB, and placed this within the wider context of understanding gap junctions between parallel axon fibres as forming an 'axonal plexus'. We believe current flows in a resistor network are a plausible interpretation of such an axonal plexus, where a sodium spike in one axon 'pulls up' and a potassium leak in another 'pulls down'. Nevertheless we accept that the model is highly abstracted and have tried to clarify that our main aim is to explore such a functional interpretation of gap junction networks, rather than the MB per se. We nevertheless believe that existing MB models have largely failed to account for the complexity of this circuit (including KC-KC connectivity) by considering only the simple associative learning and classification tasks, so the work here may be relevant to a better understanding of the MB.

For the case of the connection from the KCs to the MBONs, the proposed formula for the current isn't even realistic for a resistor network (a "current" that is a function of a voltage and a current between two other elements (two KCs) of the network). The added sentence "A possible biological interpretation of Equ 6 is that the efficacy of the chemical synapse transmission from a KC to an MBON is modulated by the gap junction connections, and consequent current flow, from that KC to other KCs." is not helping the situation as it is a highly unrealistic proposition in many ways, including because of the spiking nature of KCs.

As previously outlined, but restated in the current revision, there are several mechanisms by which gap junctions between axons in (spiking) neurons could influence the communication at their chemical synapses onto downstream neurons. E.g. the downstream neuron might require near simultaneous input from two presynaptic neurons to exceed a threshold, and this could occur when an action potential in one neuron causes an axonal action potential (independently of the soma) in the second via gap junction connectivity, a well-established phenomenon (e.g. Mercer A, Bannister AP, Thomson AM. Electrical coupling between pyramidal cells in adult cortical regions. Brain cell biology. 2006;35:13–27). This is now discussed explicitly on lines 17-21, 484-486 and lines 705-712.

I would judge the likelihood that anything resembling the mode of operation of this model is going on in insect mushroom bodies as practically zero.
Is it still useful to be inspired by the insect mushroom bodies? I believe the answer is no.

We can only reiterate that we did find it useful to be inspired by the mushroom bodies, in several ways: the idea of sparse coding of input states; the connectivity between parallel KC axons including gap junctions; the small number of action-related outputs. We accept (re comments below) these properties are not unique to MBs but do not see that as a negative.

As an additional guide, I have inspected the listed assumptions:
Assumption 1 is about sparse representations, which have been found in insect MBs. However, the model uses one-hot encoding which is a very common concept in other areas, including machine learning. I would argue, proper one-hot encoding is therefore more inspired from elsewhere.
Assumption 2 is highly unrealistic as discussed above and clearly inspired by resistor networks, not MBs.
Assumption 3 is unrealistic, given what is known about the APL/GGN in insects and inconsistent with the spiking nature of KCs.
Assumption 4 seems possible in terms of that gap junctions can be asymmetric and plastic but the information about this is not coming from MBs, so it's bio-inspired not MB-inspired.
Assumption 5 is unrealistic in terms of the role of the KC to KC current flow and otherwise just describes the idea of a read-out layer found in any neural network and in many brain models, i.e. wouldn't be a particular MB feature.
Assumption 6 refers to existing connectivity patterns in the MB but the point that synapses can be plastic and unsupervised learning can take place isn't in any way something that is particularly related to insect MBs or that MBs would be particularly known for.
In summary, none of the assumptions appear particularly MB-inspired and some of them are in contradiction with current knowledge. I don't think this makes it useful to try linking this model to insect MBs. As it is written, the title, abstract, and vast majority of the introduction are about insect mushroom bodies which are largely irrelevant to the model. I think this is very unhelpful and the changes made to the discussion are not solving this problem.
I would suggest removing insect mushroom bodies from title, abstract and introduction completely and instead discuss resistor network as an inspiration and the reinforcement learning literature that the results relate to as the main background.

See comments above. We have revised the title, abstract and introduction to reduce the emphasis on the MB, but we still believe it is still interesting to consider resistor networks as a potential interpretation of the KC-KC axonal plexus.

The Methods section is still not sufficient for a knowledgeable researcher to reproduce the model and remains confusing and imprecise. This includes the following problems (without claim of completeness of this list):
1. Appendix A is explaining how Ohm's law leads to a set of linear equations but doesn't state how these are solved (I suspect a standard algorithm from some linear algebra software package?) – however, what does "In practice, the solution converges in a few steps, which is less than the number of states." refer to?

The equations can be solved with standard linear algebra solvers, such as torch.linalg.solve() provided by PyTorch.

For a typical circuit with symmetric Ohm's law, the solution converges in one step. However, for the circuit with asymmetric connections, the solving process needs several iterations before converging. In each iteration, the potential in the last step is replaced with the new solved potential, and the equations are solved again.

2. L590: "by connecting the state node to the ground with some resistance": what resistance? Is it always the same?

The resistance varies. Smaller resistance indicates a stronger goal. It changes during learning. We have provided a parameter table (appendix C) that specifies the value used either when this connection is fixed (e.g. in the Voronoi task) or the increment with which it is changed by learning (e.g. in the Taxi task).

3. L592: "Through nodal analysis we can then establish the resulting flow of current and associate it to actions, as described in Appendix A": delete "and associate it to actions," as this is not related to either nodal analysis or Appendix A.

Deleted.

4. L604: "An activated state node represents the present state, and edges connecting the state nodes represent possible transitions between states": This is interpretation and doesn't belong in the methods. Furthermore, more generally, it remains unclear how things are done. Presumably there is an update cycle with steps including activating one state, nodal analysis, calculating a "subgoal", calculating action probabilities, choosing one of the actions, determining what the next state is? This needs to be described somewhere.

We think it adds clarity in the methods to explain how elements in the implemented system relate to their interpretation in the tasks.

See also reply 7.

5. L606: "The weight of an edge represents how often the transition happened previously": Similarly, not a description of the model.

See above, we think it would reduce clarity to remove this explanation of the edge weight interpretation (and indeed it is not just an 'interpretation', it is literally how the edge weight is set in the learning process).

See also reply 7.

6. L609: "can be updated by": What does this mean: is it updated by this equation or not?

Changed to "is updated by"

7. Equation 5: This is an ODE, how does it relate to the update sequence (point 4 above)? I am guessing that the update cycle is just discrete times steps, so how is the ODE for KC connection weights integrated? What are the timescales? What is the value of wmax?

The model is conceptually a continuous dynamic process described by differential equations. Of course, when we compute it, we adopt a numeral ODE method to approximate it, during which it was computed in discrete time steps. The numeral ODE method is simply the Euler method. In our experiment, the typical time scale is 0.02s for each step. The typical value for w_max is from 1 to 10. A paragraph has been added in the methods to describe this (lines 713-719)

Because the model selects the route relatively according to the currents or power consumption of edges, the exact value of w_max is not important, but we have indicated the choice used in particular simulations in the added table of parameters (Appendix C)

8. Throughout the Methods the references to other sections are broken, e.g. L615: "section and" … "in section ."

This appeared to be an issue with latex. The cross-references were not necessary and have been removed.

9. L623: "And although the simulated worlds we explore here are discrete, in a continuous world in which the activity of state nodes continuously changes, the connections could be built by finding negative correlations between state node activity changes": Unclear but trying to read between the lines, did they mean positive correlations between state node activations (maybe with a delay) in a Hebbian sense? (Note, also applies to L536)

Yes, conceptually similar to Hebbian learning, but using the property that the activation of one node succeeded by the another should be signalled by the decreasing activity in the first coinciding with increasing activity in the second, that is "negative correlations between state node activity changes".

With vanilla Hebbian learning, connections would be built within a group of state nodes that are active together. With Hebbian learning with delay, state nodes that are both active in two adjacent phases could become interconnected. Neither of these effects would be desirable if the aim is to capture causal state transitions.

The text has been revised for a clearer description (lines 598-603)

10. L634: "the input to connection k" -> "input to action node k"?

Corrected.

11. L644: " This could be interpreted as a lateral inhibition function between the action nodes”: While winner-take-all dynamics can be implemented with inhibition, a softmax operation is a very specific function unlikely to be easily related to simple mutual inhibition.

Reply: It is not actually essential to the method to use soft-max, we can directly use normalised I_k as the probability. In fact checking our code, although we implemented soft-max for comparison, as it potentially can increase exploration, in the results reported here we use the normalised I_k. We have revised this in the text (lines 620-626).

12. L646: “Note that as described, the operation in Equ 7 cancels out the term of power consumption in Equ 6. In this case, Equ 6 is equivalent to: …” -> while the cancellation occurs in equation (7) this does not make the stated formula valid, i.e. equation (6) is not equivalent to equation (8) only because equation (7) does not depend on “the term of power consumption”.

We agree this was not clearly worded, and have revised to make it clear that the cancellation of the power terms in equation 7 allows us to use equation 8 instead of equation 6 to determine the input to the action nodes and get the same output probability of actions (lines 627-631).

13. L651: the generalisation to multiple “subgoals” seems interesting but the formula (9) still assumes one single state i^hat contrary to the text talking about several active states.

We realise the wording here was ambiguous, and have now clarified that equation (9) relates to the situation where we do not limit the input to be only from the edge with strongest power consumption. This still uses one-hot coding for the state but multiple state nodes are selected as subgoals, so currents from several edges to action nodes are summed.Indeed, there is a further generalisation, in which sparse coding of the state is adopted, hence there will be multiple state nodes for the present state and multiple state nodes for all possible subgoals. In this case, the currents should be summed in one more level on the present state nodes, that is, summing on i^hat. We have added this point explicitly in the text (lines 631-640)

14. L673: “In the model, when a strong reward is released, the conductance between the current state and ground is set to a value that is much larger than the conductance among KCs, marking a target state for future episodes.”: to what value is it set?

In a typical setup, the default initial conductance between state nodes is 1e-10, and the default initial conductance from state nodes to ground is 1e-10, too. The maximum conductance from a state node to the ground is 1. In our experiment, with the conductance update rule that is the same as equation (5), the conductance from the target state to the ground increases from 1e-10 to 1e-3 in one step. Further learning will allow the conductance approach to 1. We have clarified this in the table of parameters (Appendix C)

15. Equation 11 and 13: please don’t use “k” both as a free index and a summation index

Revised, using \hat{K} for free index and K for summation index.

16. L721: "If the causality of task is deterministic or with little randomness, the following learning rule can be applied": what is "the causality of task"? And what does it mean that the learning rule "can" be applied? Is it applied? Always? In certain tasks? At certain times in certain tasks?

In the work here, the rule is always applied. We have modified the text (line 701-705)

17. Throughout, e.g. L725: "with a typical value of …": What does this mean? Is the value as stated? Or is it different in the different experiments? If the latter, please state the value for each of the experiments separately.

We have now clarified the actual values used (line 701)

18. Please also state the values for all other parameters such as learning rates alpha etc

A table of parameters has been added to the appendix.

Reviewer #2:

The authors have fully addressed my previous comments.

Thankyou.

Reviewer #3:

Overview

The authors have made excellent improvements to the flow and readability of the manuscript.

Thankyou.

They have introduced a new results section, applying their dynamic routing model to a typical conditioning experiment performed with insects. Unfortunately, this section introduces several new sources of confusion that would benefit from clarification.

We have tried to rewrite this section (lines 287-363) to remove confusion, as detailed below.

Major Comments

Simple associative learning – there are quite a few ambiguities in this application of the model, and as a result I am finding it very difficult to make sense of it. I appreciate that the

authors are trying to be concise, but I think some details need to be stated explicitly, and others would benefit from rewording to make the explanations more clear.

We have revised this section to try to make it clearer, including simplifying the example to positive reinforcement only.

1. It's not clear to me why, in the test phase without reinforcement, the maggot keeps selecting the previously reinforced odour, but does so much less when there is reinforcement in the test phase. E.g., take training protocol 1 (training with AM+/OCT and test petri dish AON). During training, state transition state1→state4 is eventually selected with action "toward AM", resulting in that state transition and that action being strengthened. The stronger it becomes, the more likely it is selected, and so it becomes even stronger. Then, in the test phase, I presume the state1→state4 connection has the max power (though this needs to be stated explicitly if so, as it is not obviously the case when state 4 is no longer grounded), and the "toward AM" action continues to be chosen. However, because this action no longer results in the maggot occupying state 4, but rather state 1 again (as there is no reward), the "toward AM" action is weakened, making it less likely to be chosen again. Maybe the action's weight is so strong that this doesn't matter, but after 40 steps I would have thought it would become significantly weaker. Either way, the consequences need to be explained as it's not obvious how the model will evolve (diagrams and figures showing the evolution of action weights and state connection weights etc. would be a great help). Conversely, in protocol 3, when there is reinforcement during the test phase (petri dish AOF), state 4 is grounded (or as before, the state1→state4 weight is large), giving that connection high power, and the action "select AM" results in the maggot occupying state 4, thus further strengthening the action. At least, that is how I understand it.

We have added supplementary figures (fig 17 and 18) showing the evolution of the weights. Note that there is no mechanism to weaken connections between states, or from states to ground, and during test state 4 (fructose) remains a target (connected to ground). The connections from state transitions to actions can be weakened, and in the 40 steps of the test without fructose, there is weakening of the action 'appetitive to AM' but it still remains stronger than any alternative action. In the test with fructose, the action is further strengthened but the competing action 'appetitive to OCT' also becomes strengthened so the choice 'towards AM' becomes less frequent. We have revised the explanation to make this clear (lines 345-363).

2. Maybe this is related to point 1 above, but it's not clear to me what happens when the maggot moves to state 4. Is it automatically moved back to state 1 after the reward is received, as it is in the presence of odour AM, after all. Or does it then have to move from state 4 to another state in the next step? If the latter, and it selects "toward AM" again, is the maggot kept in state 4 (thus weakening the action "toward AM" because it stems from connections to anywhere but state 4), or does it move to state 1, allowing the action to be strengthened?

The maggot can remain in the same state if the selected action results in it occupying the same state (e.g. if in state 4, it takes 'appetitive to AM' action that results in it being in a location that contains AM and fructose). However (see next), there are no self-connections so no 'edge' connecting the state to itself, and thus no possibility to associate an action to staying in the same state.

3. If it is indeed possible for the maggot to remain in the same state over consecutive steps, are self-connections therefore incorporated in this paradigm?

Reply: No, there are no self-connections.

4. If the maggot is in state 1 (AM present), there is presumably no odour gradient. Are actions always random in this case, even if there is OCT elsewhere in the petri dish? I.e. is an odour gradient only possible in petri dishes with both odours AND when the maggot is in state 3? Or is the maggot aware of the presence of an odour gradient in AON/AOF/AOG dishes, even when it is not actually in a gradient containing state? Whichever it is, it needs explaining explicitly.

The gradients extend across the whole dish when both odours are present, that is, AM gets stronger from right to left, and OCT from left to right, so the maggot is aware of both gradients in every position when it takes a directional action. We have updated the description to make this clearer (lines 302-305)

5. Line 337-339 – Take the AM+/OCT training protocol. Am I right in thinking that the agent only receives fructose reward if it is on an AM state, and then approaches the other AM state? As though a maggot has to crawl to the far end of a corridor before it receives a reward. If so, could the sentence starting "When a maggot takes an appetitive action…" be rephrased to make this more explicit.

The 'appetitive to AM' action needs to result in the agent entering a location with AM (that also contains fructose) for fructose to be experienced (and similarly for appetitive to OCT). We have clarified this in the text (lines 310-313).

6. Line 373 – Is the fact that there is no current flow because the high potential due to being in state 4 is countered by the grounded potential due to the presence of reward? This needs a more clear explanation.

Yes, we have made this clearer on lines 356-360.

7. Table 1 – During testing, for petri dishes with two odours and a reinforcement, it needs to be stated which odour receives the reinforcement. Initially, I presumed it was the same as the odour that received reinforcement during training, but lines 379-381 make me think that perhaps both odours can provide the same reinforcement. Either way, this needs to be made explicit.

Two odours and a reinforcer occurs only during testing, in which case both odours receive the reinforcement (if the maggot does the appropriate action with respect to that odour).

8. It's not clear to me when the default state is activated, if ever.

Reply: In the paradigms explored here, the default state is indeed never used, we have noted this in the text (lines 318-319) but left it in the description for generality.

Minor Comments

9. Table 5 – “appetite” and “averse” maybe better replaced with “toward” and “escape”, to be consistent with language used previously.

We have instead used “appetitive” and “aversive” throughout, and not “toward” or “escape”.

10. Line 646-647 – It’s stated that Eq. 6 is equivalent to some expression given in Eq. 8, but I don’t think this is so. I think Eq. 6 should always depend on the power consumption. It is the term inside the parenthesis in Eq. 7 that is equivalent to Eq. 8, as that is where the power is cancelled out. Thus, the term inside the parenthesis is equivalent to c_1 * w_ijk / max_k(w_ijk)

Reply: We agree this was not clearly worded, and have revised to make it clear that the cancellation of the power terms in equation 7 allows us to use equation 8 instead of equation 6 to determine the input to the action nodes and get the same output probability of actions (lines 627-631).

11. Line 368 – “state 3 (fructose)” seems incorrect. Fructose is state 4.

Fixed

12. Line 603 – It is not clear what “Assumption 1” is, but interpreting the rest of the sentence depends on it.

The overall revision of the text means this comment no longer applies.

13. Line 657-662 – I think some additional explanation would be helpful here, especially in terms of pointing out the caveats of this interpretation. In particular – and if I understand the mechanisms properly – the biological interpretation that has been given would make more sense if all connections emanating from a particular node contributed to the current received by a particular action, rather than the current being a function of the connection to the node with the highest power consumption only. The approximation, ie. whereby only the connection to the highest power node drove the action, would then make sense if the inverse temperature in the softmax function was very high.

We have revised this part of the discussion (lines 661-667) and hope the proposed mechanism is now clear.

14. Line 157 – it is a bit confusing to say there are 3 dimensions, when each

    We rephrased to say three factors determine the dimensions (line 143)

15. RE minor comment 15 in my previous review – Regarding the selection of a connection to the highest power node, I believe a clear explanation of why this is a local operation is needed. I don’t yet see how it is local, as the synaptic

connection itself doesn't have local access to the necessary information needed to effectively gate open/closed "second-order" connections to the action nodes. Even though the connections share a common neuron (that of the current state), I don't yet see how information about the potentials at connected nodes is shared between connections. In a standard neural circuit model, it would be unusual for a synapse between neuron A and B to be silenced by a stronger synapse between A and C, apart from when some normalisation is used after a weight update, which itself is a nonlocal operation, and in any case is not the mechanism at play here.

It is not actually crucial to the model that only the strongest connection from the active node is considered. Allowing all edges from the current node to contribute input to actions according to the current flow should result in a similar action choice, which corresponds to a more general setup in that an action is chosen by a weighted sum of the contribution of every subgoal. The intention of the model is to introduce the overall mechanism and demonstrate its functionality, so we chose the simpler version. Alternatively, as in our clarified discussion of how downstream action might be understood biologically, it could be that the strongest gap junction connection is the only one by which sufficient current is passed to cause an axonal action potential and hence simultaneous synaptic release from the two connected axons.

16. RE minor comment 30 in my previous review – I didn't properly understand the new caption for Fig 5 until I read the authors' response to my comment. I think the caption should be replaced with the authors' response to clearly explain what is going on.

Revised as suggested (this is now figure 4)

17. Line 258 – It states that edges without walls are the passable edges, but also that every edge crosses a wall, suggesting that, in fact, no edges are passable. I think it might be easier to follow if the construction of the Voronoi world is described in this order: 1) Voronoi walls are constructed, 2) Delauney triangulation constructed, 3) a random selection of Voronoi walls are removed, 4) corresponding Delauney edges are removed, leaving only edges that an agent can follow around the walls.

Revised as suggested (line 232-243)

18. Fig. 8 – Can numbers be added to panel B to aid comparison between A and B?

Reply: The plot was generated with a random number generator when the experiment was executed, so it is a bit difficult to plot the exact same map again. However, Fig 8 (a) (note this was fig 9) has the same map with numbered locations. For a more convenient comparison, we illustrated available paths in Fig 7 (b) (note this was fig 8).

19. Fig. 9 – there is some spurious information in panel A, "step 14 | Fuel left: 9987 | Rewards: 19", which doesn't seem relevant.

The text has been removed (note this is now fig 8).
